# Genetic Determinants of Resistance to Extended-Spectrum Cephalosporin and Fluoroquinolone in Escherichia coli Isolated from Diseased Pigs in the United States

**DOI:** 10.1128/mSphere.00990-20

**Published:** 2020-10-28

**Authors:** Shivdeep Singh Hayer, Seunghyun Lim, Samuel Hong, Ehud Elnekave, Timothy Johnson, Albert Rovira, Fabio Vannucci, Jonathan B. Clayton, Andres Perez, Julio Alvarez

**Affiliations:** a Department of Veterinary Population Medicine, College of Veterinary Medicine, University of Minnesota, Saint Paul, Minnesota, USA; b Department of Biology, University of Nebraska at Omaha, Omaha, Nebraska, USA; c Bioinformatics and Computational Biology Program, University of Minnesota—Rochester, Rochester, Minnesota, USA; d Department of Microbiology and Immunology, Rega Institute, KU Leuven, Leuven, Belgium; e Department of Veterinary and Biomedical Sciences, College of Veterinary Medicine, University of Minnesota, Saint Paul, Minnesota, USA; f Veterinary Diagnostic Laboratory, College of Veterinary Medicine, University of Minnesota, Saint Paul, Minnesota, USA; g Nebraska Food for Health Center, University of Nebraska—Lincoln, Lincoln, Nebraska, USA; h VISAVET Health Surveillance Center, Universidad Complutense Madrid, Madrid, Spain; i Department of Animal Health, Facultad de Veterinaria, Universidad Complutense Madrid, Madrid, Spain; Antimicrobial Development Specialists, LLC

**Keywords:** plasmids, ESBLs, swine, USA, PMQR, WGS, cephalosporin, fluoroquinolone, antimicrobial resistance, PacBio, long-read sequencing, high-risk clones, epidemic plasmids

## Abstract

Fluoroquinolones and cephalosporins are critically important antimicrobial classes for both human and veterinary medicine. We previously found a drastic increase in enrofloxacin resistance in clinical Escherichia coli isolates collected from diseased pigs from the United States over 10 years (2006 to 2016). However, the genetic determinants responsible for this increase have yet to be determined. The aim of the present study was to identify and characterize the genetic basis of resistance against fluoroquinolones (enrofloxacin) and extended-spectrum cephalosporins (ceftiofur) in swine E. coli isolates using whole-genome sequencing (WGS). *bla*_CMY-2_ (carried by IncA/C2, IncI1, and IncI2 plasmids), *bla*_CTX-M_ (carried by IncF, IncHI2, and IncN plasmids), and *bla*_SHV-12_ (carried by IncHI2 plasmids) genes were present in 87 (82.1%), 19 (17.9%), and 3 (2.83%) of the 106 ceftiofur-resistant isolates, respectively. Of the 110 enrofloxacin-resistant isolates, 90 (81.8%) had chromosomal mutations in *gyrA*, *gyrB*, *parA*, and *parC* genes. Plasmid-mediated quinolone resistance genes [*qnrB77*, *qnrB2*, *qnrS1*, *qnrS2*, and *aac-(6)-lb′-cr*] borne on ColE, IncQ2, IncN, IncF, and IncHI2 plasmids were present in 24 (21.8%) of the enrofloxacin-resistant isolates. Virulent IncF plasmids present in swine E. coli isolates were highly similar to epidemic plasmids identified globally. High-risk E. coli clones, such as ST744, ST457, ST131, ST69, ST10, ST73, ST410, ST12, ST127, ST167, ST58, ST88, ST617, ST23, etc., were also found in the U.S. swine population. Additionally, the colistin resistance gene (*mcr-9*) was present in several isolates. This study adds valuable information regarding resistance to critical antimicrobials with implications for both animal and human health.

**IMPORTANCE** Understanding the genetic mechanisms conferring resistance is critical to design informed control and preventive measures, particularly when involving critically important antimicrobial classes such as extended-spectrum cephalosporins and fluoroquinolones. The genetic determinants of extended-spectrum cephalosporin and fluoroquinolone resistance were highly diverse, with multiple plasmids, insertion sequences, and genes playing key roles in mediating resistance in swine Escherichia coli. Plasmids assembled in this study are known to be disseminated globally in both human and animal populations and environmental samples, and E. coli in pigs might be part of a global reservoir of key antimicrobial resistance (AMR) elements. Virulent plasmids found in this study have been shown to confer fitness advantages to pathogenic E. coli strains. The presence of international, high-risk zoonotic clones provides worrisome evidence that resistance in swine isolates may have indirect public health implications, and the swine population as a reservoir for these high-risk clones should be continuously monitored.

## INTRODUCTION

Antimicrobial resistance has emerged as an issue of grave concern in both human and veterinary medicine. Food animals are considered potential reservoirs of antimicrobial-resistant and zoonotic pathogens such as Escherichia coli, although the extent of spread of resistant bacteria via the food chain is still under debate (1). Critically important antimicrobials for human medicine such as cephalosporins and fluoroquinolones are still used in many parts of the world to treat diseased food animals, including swine in the United States (2–4). Furthermore, certain genetic determinants responsible for resistance to antimicrobials approved for use in animals (such as ceftiofur and enrofloxacin) and those used in human medicine (such as cefoxitin and ciprofloxacin) are the same (5, 6). It is therefore important to monitor the circulation of genes responsible for resistance to such critically important antimicrobials in bacteria present in humans and animals to develop better source attribution models and targeted interventions in both human and veterinary medicine (7). A recent ban on colistin use in animal agriculture in China is an example of surveillance of antimicrobial resistance genes leading to actual policy changes (8) and a decrease in colistin resistance (9).

Resistance to extended-spectrum cephalosporins is complex and mediated by extended-spectrum beta-lactamases (ESBLs) (commonly encoded by the *bla*_TEM_, *bla*_SHV_, and *bla*_CTX-M_ genes), carbapenemases (encoded by *bla*_KPC_, *bla*_NDM_, *bla*_OXA-48_, etc.), plasmidic AmpC (pAmpC; commonly encoded by the *bla*_CMY_ genes), and mutations in AmpC promoter regions in the chromosome (10, 11). These *bla* genes may be inserted on bacterial chromosomes but are usually present on plasmids with the potential to disseminate horizontally to other bacterial strains (12). *bla*_CTX-M_ genes are reported as the most prevalent ESBL genes worldwide in humans and animals (13). However, *bla*_CMY-2_ genes were primarily responsible for extended-spectrum cephalosporin resistance in bacteria of food animal origin in North America, while other ESBL-encoding genes were not reported until the late 2000s (14). Nevertheless, recent reports have also suggested the emergence of ESBL genes in bacteria of food animal origin in teh United States over the last decade (15). So far, *bla*_CMY-2_ genes have been found to be present on IncA/C2 and IncI1 plasmids in farm animals both globally (16–19) and in the United States (20, 21). On the other hand, *bla*_CTX-M_ genes have been found to be present on IncF, IncI1, and IncN in farm animals in the United States (15, 22, 23).

Resistance to fluoroquinolones is mainly mediated by multiple chromosomal mutations in certain genes (*gyrA*, *gyrB*, *parE*, and *parC*). Additionally, plasmid-mediated quinolone resistance genes (such as *qnr*) and upregulation of efflux pumps confer variable levels of resistance to this antimicrobial family (24). *qnr* genes encoded in plasmids were also found in *Salmonella* isolates collected from retail pork, cecal samples from healthy pigs, and clinical samples from diseased pigs in the same period, suggesting a likely role in the increase in phenotypic resistance (25–27). An increase in fluoroquinolone resistance was recently reported in Salmonella enterica isolates from diseased pigs in Minnesota between 2006 and 2015 (2). A similar increase in phenotypic resistance to a fluoroquinolone (enrofloxacin) was also reported for the same time frame in swine E. coli clinical isolates (28), though the genetic determinants mediating this increase have not been determined yet.

Selective pressure due to exposure to antimicrobials or other chemicals can lead to a quick emergence of resistant bacterial strains (29). Clones of these resistant strains can quickly disseminate through the populations if they have key fitness advantages over nonresistant strains (29). In some instances, the presence of additional virulence factors can also confer additional fitness advantage on these bacterial clones. A hallmark example of clonal dissemination of a dominant E. coli clone is the global emergence of the highly pathogenic E. coli ST131 lineage that has been associated with the acquisition of mutations in quinolone resistance-determining genes or virulent IncF epidemic plasmids carrying *bla*_CTX-M_ genes (30).

Although increasing information on the prevalence of phenotypic resistance in bacteria (including E. coli) of animal origin is generated by national antimicrobial resistance (AMR) monitoring programs such as NARMS (31), there is limited information on the genetic backbone mediating these resistance phenotypes. This may be of particular importance in the case of critically important antimicrobials such as fluoroquinolones, cephalosporins, or carbapenems. The objective of this study was to characterize the genetic basis of fluoroquinolone and extended-spectrum cephalosporin resistance in phenotypically resistant E. coli isolates collected from diseased pigs in the United States between 2014 and 2015 using both short-read (Illumina) and long-read (PacBio) whole-genome sequencing (WGS).

## RESULTS

### Genetic determinants conferring extended-spectrum cephalosporin and fluoroquinolone resistance.

**FIG 1:**
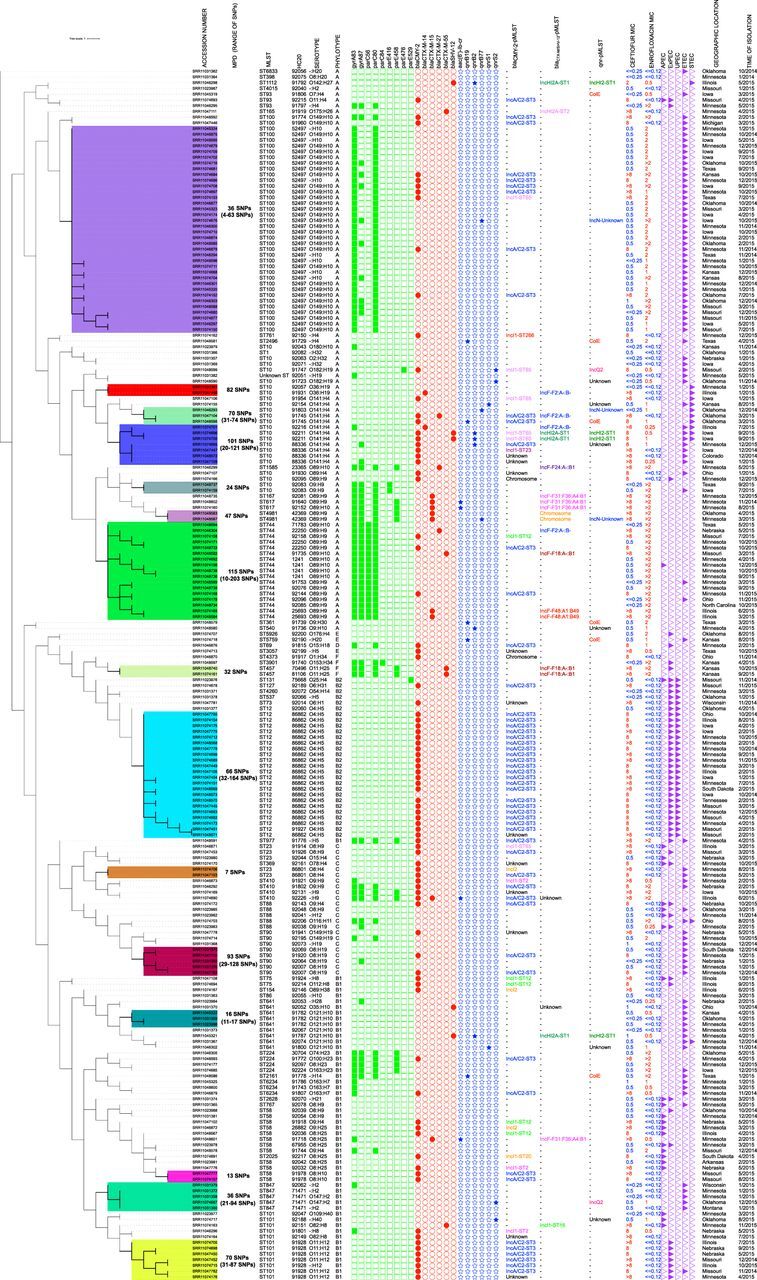
Maximum likelihood tree constructed using the core gene alignment of Escherichia coli isolates collected from diseased pigs at UMN-VDL between 2014 and 2015. This unrooted tree was created by mapping raw reads on E. coli K-12 substrain MG1655 (accession NZ_AJGD00000000.1), followed by extraction of SNPs and phylogenetic tree construction (GTR plus gamma substitution model, 1,000 bootstrap replicates) using RAxML version 8.0. Raw reads mapped onto 84.1% to 94.9% of the reference sequence E. coli K-12 substrain MG1655. Phylogenetic tree was constructed using 100,569 recombinant-free sites. Ceftiofur and enrofloxacin MIC values (in μg/ml) are labeled in red and blue to denote resistant and susceptible isolates, respectively. Heat map shows presence of chromosomal mutations in quinolone resistance-determining regions (QRDRs) (green squares), plasmid-mediated quinolone resistance (PMQR) genes (blue stars), ESBL/pAmpC genes (red circles), and virulotypes (APEC, ExPEC, UPEC, ETEC, and STEC) (purple triangles). Median pairwise SNP distances (MPDs) were estimated using ST-specific references. Colored clusters represent groups of isolates with an SNP distance to the next closest isolate of less than 100. Unknown, plasmids/chromosomes carrying these genes were not identified for these isolates.

Of 106 ceftiofur-resistant isolates, 87 (82.1%) carried *bla*_CMY-2_ genes (Fig. 1). These genes were not present in the remaining 105 ceftiofur-susceptible isolates. Isolates carrying this gene belonged to 24 different sequence types (STs), with ST12 (*n* = 21) and ST101 (*n* = 10) being the dominant STs (Fig. 1). Nineteen isolates of 11 different STs carried *bla*_CTX-M_ genes (Fig. 1). All of the 19 *bla*_CTX-M_-carrying isolates were ceftiofur resistant. Five isolates of 3 different STs carried the *bla*_SHV-12_ gene, and 2 of these five isolates were ceftiofur susceptible. Twenty-two of the 106 ceftiofur-resistant isolates carried *bla*_CTX-M_ or *bla*_SHV-12_ genes, whereas only 2 of the 105 ceftiofur-susceptible isolates carried *bla*_SHV-12_ genes. Four isolates carried combinations of *bla*_CMY-2_-*bla*_CTX-M_ or *bla*_CMY-2_-*bla*_SHV-12_ genes.

Multiple fluoroquinolone resistance-associated genes and mutations were detected in 106 of the 110 enrofloxacin-resistant E. coli isolates (Table 1; see also Table S1 in the supplemental material), while only four of the 101 susceptible isolates presented any of them (specifically, single mutations in the *gyrA* gene [S83L or D87Y]). Isolates resistant to enrofloxacin belonged to 30 different STs.

**TABLE 1:** Pattern of genetic determinants of enrofloxacin resistance in E. coli clinical isolates of swine origin

MIC (μg/ml)	Pattern of genetic determinants (no. of isolates)	ST type(s) (no. of isolates)
>2	*gyrA*(S83L) + *gyrA*(D87Y or D87N or D87G) + *parC*(S80I or S80R) ± other genetic determinants^*a*^ (49)	744 (11), 100 (10), 224 (4), 410 (3), 10 (2), 457 (2), 617 (2), 4981 (2), 88 (1), 93 (1), 167 (1), 977 (1), 1585 (1), 2161 (1), 3901 (1)
2	*gyrA*(S83L) + *parC*(S80I or S80R) (23)	100 (21), 58 (1), 90 (1)
	*qnrB19* (2)	361 (1), 2496 (1)
	No genetic determinants (1)	5926 (1)
1	*gyrA*(S83L) + *parC*(S80I or S80R) (7)	100 (6), 69 (1)
	*gyrA*(S83L) only (1)	6234 (1)
	*gyrA*(D87G) + *qnrB2* (1)	10 (1)
	*aac(6′)-Ib-cr* + *qnrB2* (1)	540 (1)
	Single PMQR (*qnrB19*, *qnrS1*, *qnrS2*, *qnrB2*, or *qnrB77*) (8)	10 (4), 101 (1), 641 (1), 847 (1), 5759 (1)
0.25–0.5	*gyrA*(S83L) ± *aac(6′)-Ib-cr* (6)	6234 (2), 10 (1), 58 (1), 101 (1), 410 (1)
	Single PMQR (*qnrB19*, *qnrS1, qnrS2*, *qnrB2*) (5)	10 (3), 93 (1), 1112 (1)
	*gyrA* (D87G or D87Y) (3)	10 (1), 88 (1), 641 (1)
	*aac(6′)-Ib-cr* + *qnrB2* (1)	641 (1)
	No genetic determinants (2)	641 (1), 3057 (1)
≤0.125	*gyrA*(S83L) (3)	10 (1), 847 (1), unknown (1)
	*gyrA*(D87Y) (1)	90 (1)

a This genetic determinant might or might not be present in isolates with that particular MIC value. Other determinants were *parC*(A56T or E84G), *parE*(S458A or L416F), and single PMQR [*aac(6′)-Ib-cr*, *qnrB77*, or *qnrB19*].

10.1128/mSphere.00990-20.1 TABLE S1 Detailed virulence, antimicrobial resistance, and typing metadata of all isolates generated in this study. Download Table S1, XLSX file, 0.2 MB. Copyright © 2020 Hayer et al. 2020 Hayer et al. This content is distributed under the terms of the Creative Commons Attribution 4.0 International license.

Six different types of plasmid-mediated quinolone resistance (PMQR) genes were identified in a total of 25 isolates spread across 7 states (Fig. 1; Table S1). These 25 isolates belonged to 16 different STs (Table 1, Fig. 1, and Table S1). Enrofloxacin MIC values for isolates with a single PMQR gene, two PMQR genes, and one PMQR gene plus a chromosomal mutation (*gyrA*-S83L, D87G, or *parE*-D476A) ranged between 0.5 and 1.0 μg/ml, with the exception of two isolates that carried only *qnrB19* but had an enrofloxacin MIC value of 2 μg/ml.

### Description of assembled plasmids carrying PMQR and ESBL genes.

**FIG 2:**
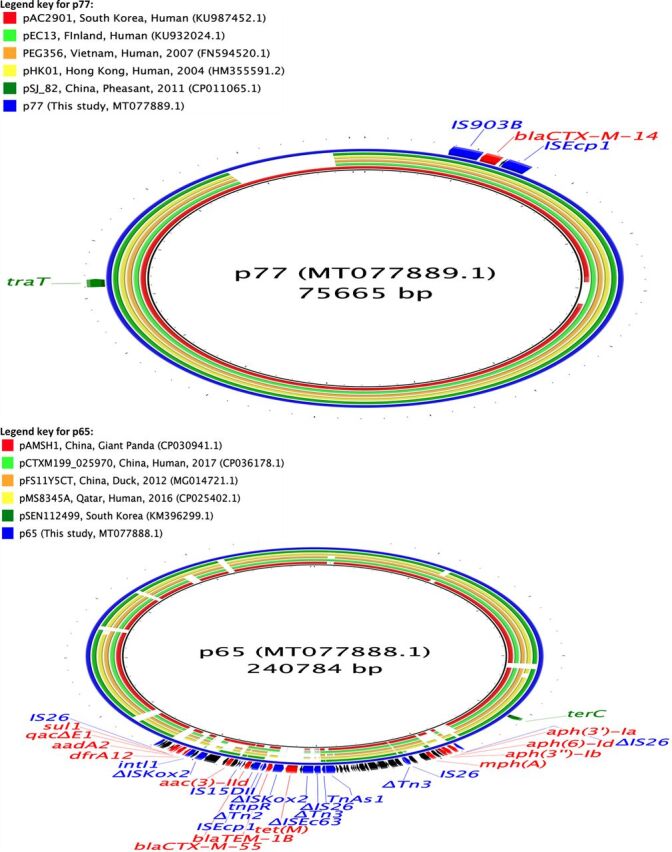
Circular maps representing comparisons of *bla*_CTX-M-14_ (p77)- and *bla*_CTX-M-55_ (p65)-carrying plasmids available at GenBank and plasmids assembled in this study. The innermost rings (not colored black) represent the top plasmids with high nucleotide identity and coverage with respect to reference plasmids (p77 and p65). The legends at the top left present plasmid name, country, animal species/human, and year of isolation, where available. Areas of the plasmids carrying AMR genes are presented in the outermost rings. AMR genes, genes associated with mobile elements, and virulence genes are colored and labeled in red, blue, and green, respectively. Truncated genes are presented with Δ as a prefix.

**FIG 3:**
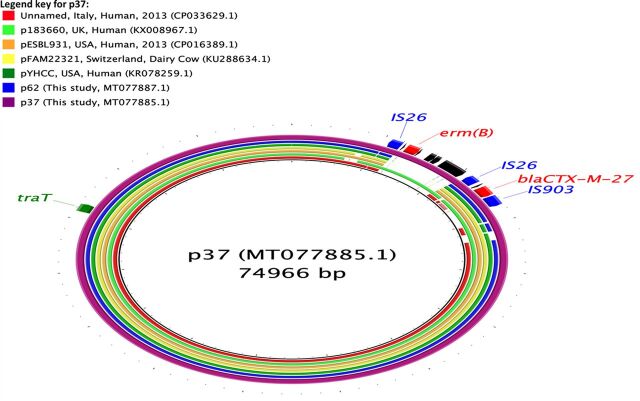
Circular map representing comparison of *bla*_CTX-M-27_ (p37 and p62)-carrying plasmids available at GenBank and plasmids assembled in this study. The innermost rings (not colored black) represent the top plasmids with high nucleotide identity and coverage with respect to reference plasmid (p37). The legend at the top left presents plasmid name, country, animal species/human, and year of isolation, where available. Area of the plasmid carrying AMR genes is presented in the outermost ring. AMR genes, genes associated with mobile elements, and virulence genes are colored and labeled in red, blue, and green, respectively.

**FIG 4:**
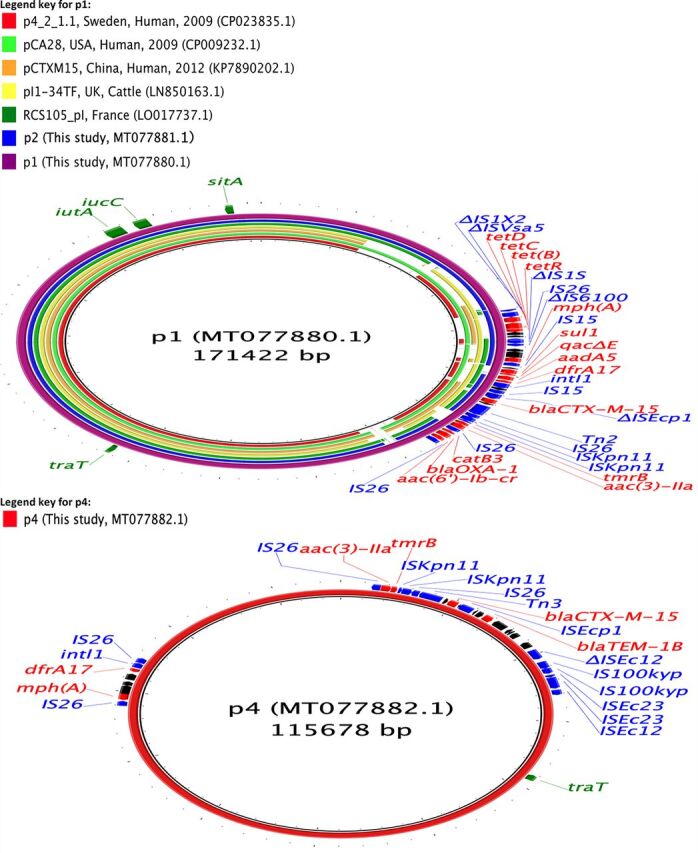
Circular maps representing comparisons of *bla*_CTX-M-15_ (p1, p2, and p4)-carrying plasmids available at GenBank and plasmids assembled in this study. The innermost rings (not colored black) represent the top plasmids with high nucleotide identity and coverage with respect to reference plasmids (p1). There were no plasmids similar to p4. The legends at the top left present plasmid name, country, animal species/human, and year of isolation, where available. Areas of the plasmids carrying AMR genes are presented in outermost rings. AMR genes, genes associated with mobile elements, and virulence genes are colored and labeled in red, blue, and green, respectively. Truncated genes are presented with Δ as a prefix.

We assembled complete E. coli chromosome and plasmid sequences using both long and short reads from 10 isolates (six isolates carrying only *bla*_CTX-M_ genes, two carrying *bla*_SHV-12_ genes, one carrying *bla*_CTX-M_ and *qnrB77* genes, and one carrying *bla*_CTX-M_ and *bla*_CMY-2_ genes) (Table 2). In seven of the isolates, *bla*_CTX-M_ genes were present on IncFII (*bla*_CTX-M-14, -15, -27_) and IncHI2 (*bla*_CTX-M-55_) plasmids, with sizes ranging between 69 and 240 kbp. *bla*_CTX-M_ genes were present in regions flanked by IS*26*, IS*Ecp1*, IS*5*, IS*6*, and Tn*3* family transposases, which were often truncated (Table 2; Fig. 2, 3, and 4). In one isolate, *bla*_CTX-M-15_ was present on the E. coli chromosome, flanked by transposases similar to those surrounding *bla*_CTX-M-15_ in the IncFII plasmids. Plasmids with *bla*_CTX-M-14_ or *bla*_CTX-M-27_ carried only *bla*_CTX-M_ or one other AMR gene [*erm(B)*, a macrolide resistance gene], whereas the plasmids carrying *bla*_CTX-M-15_ and *bla*_CTX-M-55_ also bore genes which can confer resistance to aminoglycosides, penicillins, macrolides, or trimethoprim (Table 2; Fig. 2 to 4). Additionally, some of these *bla*_CTX-M-15_ and *bla*_CTX-M-55_ plasmids also carried genes that can cause resistance to sulfonamides, phenicols, or tetracyclines (Table 2; Fig. 2 and 4). Two of the *bla*_CTX-M-15_-carrying IncFII plasmids also harbored the *aac(6′)-Ib-cr* gene which can confer resistance to both aminoglycosides and fluoroquinolones (Table 2; Fig. 4).

**TABLE 2:** Characteristics of plasmids assembled in this study

Plasmid (GenBank accession no.)	Gene of interest	Size of plasmid (kbp)	Replicon type (pMLST)	ST	Other AMR gene(s) [drug resistance]^*a*^	Virulence gene(s)
p77 (MT077889)	*bla*_CTX-M-14_	76	IncF (F2:A-:B-)	10		*traT*
p37 (MT077885)	*bla*_CTX-M-27_	75	IncF (F2:A-:B-)	744	*erm(B)* [MA]	*traT*
p62 (MT077887)	*bla*_CTX-M-27_	69	IncF (F2:A-:B-)	10		*traT*
p1 (MT077880)	*bla*_CTX-M-15_	171	IncF (F31:F36:A4:B1)	617	*aadA5*, *aac(3)-IIa*, *aac(6′)-Ib-cr* [AM]; *bla*_OXA-1_ [PE]; *mph(A)* [MA]; *sul1*, *dfrA17* [TS]; *catB3* [PH]; and *tet(B)* [TE]	*traT*, *sitA*, *iucC*, *iutA*
p2 (MT077881)	*bla*_CTX-M-15_	168	IncF (F31:F36:A4:B1)	58	*aac(6′)-Ib-cr* [AM]; *bla*_OXA-1_ [PE]; *mph(A)* [MA]; *dfrA17* [TS]; *catB3* [PH]; and *tet(B)* [TE]	*traT*, *sitA*, *iucC*, *iutA*
p4 (MT077882)	*bla*_CTX-M-15_	115	IncF (F48:A1:B49)	744	*aac(3)-IIa* [AM]; *bla*_TEM-1b_ [PE]; *mph(A)* [MA]; and *dfrA17* [TS]	*traT*
p65 (MT077888)	*bla*_CTX-M-55_	241	IncHI2 (ST-2)	165	*aac(3)-IId*, *aadA2*, *aph(3″)-Ib*, *aph(3′)-Ia*, *aph(6)-Id* [AM]; *bla*_TEM-1b_ [PE]; *mph(A)* [MA]; *sul1*, *dfrA12* [TS]; and *tet(M)* [TE]	*terC*
p33 (MT077884)	*bla*_SHV-12_	302	IncHI2 (ST-1)	641	*aac(6′)-Ib3*, *aac(6′)-IIc*, *aph(6′)-Id*, *aph(3′)-Ib*, *aadA2*, *aac(6′)-Ib-cr* [AM]; *bla*_TEM-1b_ [PE]; *qnrB2* [FL]; *ere(A)* [MA]; *sul1*, *sul2*, *dfrA19* [TS]; *tet(D)* [TE]; and *mcr-9* [CO]	*terC*
p39 (MT077886)	*bla*_SHV-12_	289	IncHI2 (ST-1)	1112	*aph(3″)-Ib*, *aph(6′)-Id*, *aph(3′)-Ia*, *aac(6′)-IIc* [AM]; *bla*_TEM-1b_ [PE]; *ere(A)* [MA]; *sul1* [TS]; *catA2* [PH]; *tet(D)* [TE]; and *mcr-9* [CO]	*terC*
pCMY (MT816498)	*bla*_CMY-2_	168	IncA/C2 (ST-3)	10	*aac(3)-VIa*, *aadA24*, *aph(3″)-Ib*, *aph(6)-Id* [PE]; *sul1*, *sul2* [TS]; *floR* [PH]; and *tet(A)* [TE]	
p23 (MT077883)	*qnrB77*	59	IncN (unknown)	4981	*aac(3)-VIa*, *aadA1* [AM]; and *dfrA15* [TS]	

a AM, aminoglycosides; PE, penicillins; FL, fluoroquinolones; MA, macrolides; TS, trimethoprim/sulfonamide; PH, phenicols; TE, tetracyclines; CO, colistin.

**FIG 5:**
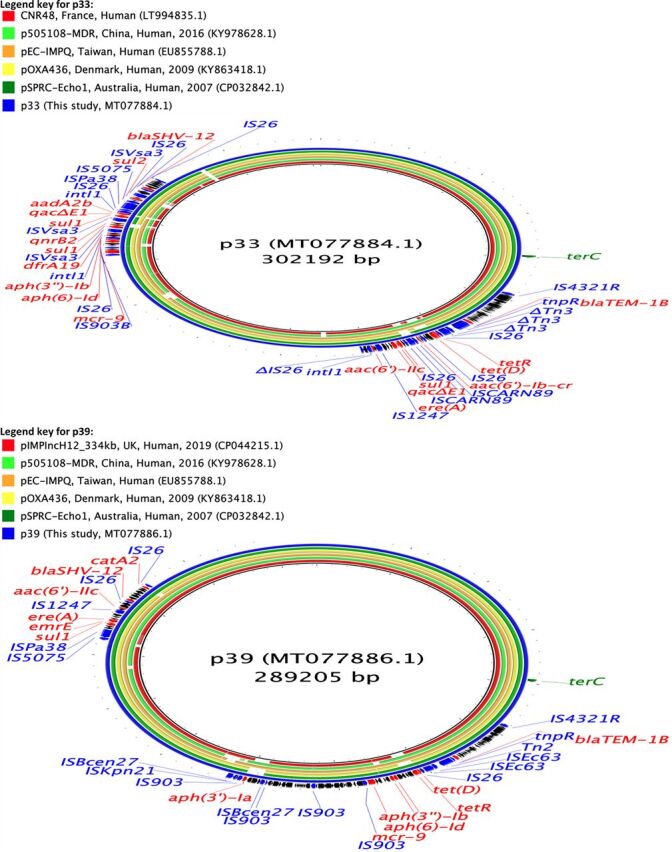
Circular maps representing comparisons of *bla*_SHV-12_ (p33 and p39)-carrying plasmids available at GenBank and plasmids assembled in this study. The innermost rings (not colored black) represent the top plasmids with high nucleotide identity and coverage with respect to reference plasmids (p33 and p39). The legends at the top left present plasmid name, country, animal species/human, and year of isolation, where available. Areas of the plasmids carrying AMR genes are presented in the outermost rings. AMR genes, genes associated with mobile elements, and virulence genes are colored and labeled in red, blue, and green, respectively.

The two plasmids carrying *bla*_SHV-12_ genes assembled were of large IncHI2-type plasmids (approximately 287 to 300 kbp) and carried genes for resistance to aminoglycosides, sulfonamides, trimethoprim, tetracyclines, penicillins, phenicols (only p39), and macrolides (Table 2; Fig. 5). *bla*_SHV-12_ genes were present in a region flanked by intact IS*6* family transposases. One of these plasmids also carried genes for resistance to fluoroquinolones [*qnrB2*, *aac(6′)-Ib-cr*] and both of these plasmids also carried a colistin resistance gene (*mcr-9*) (Table 2; Fig. 5).

**FIG 6:**
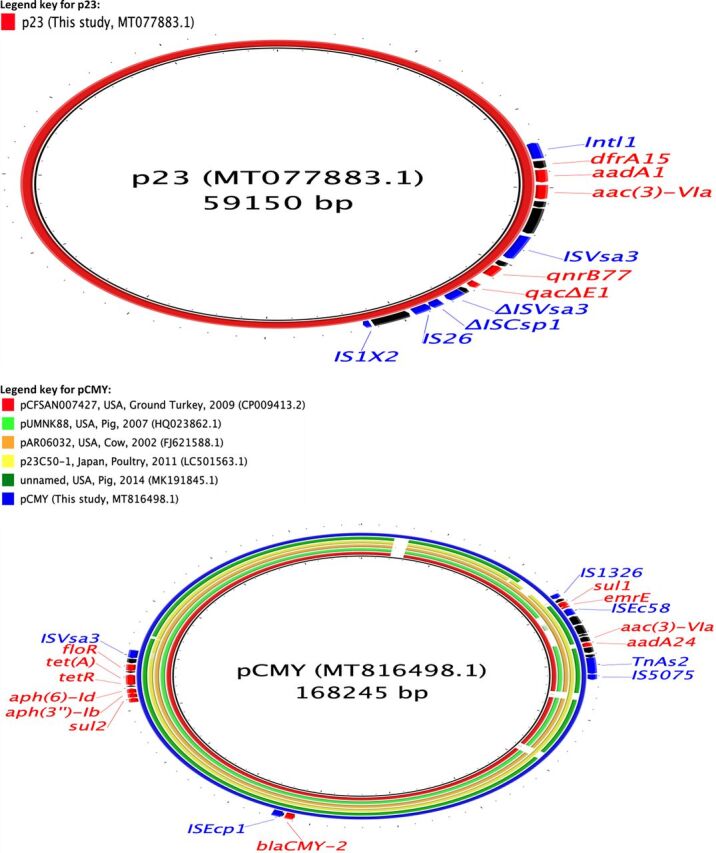
Circular maps representing comparisons of *qnrB77* (p23)- and *bla*_CMY-2_ (pCMY)-carrying plasmids available at GenBank and plasmids assembled in this study. The innermost rings (not colored black) represent the top plasmids with high nucleotide identity and coverage with respect to reference plasmids (pCMY). There were no plasmids similar to p23. The legends at the top left present plasmid name, country, animal species/human, and year of isolation, where available. Areas of the plasmids carrying AMR genes are presented in the outermost rings. AMR genes and genes associated with mobile elements are colored and labeled in red and blue, respectively. Truncated genes are presented with Δ as a prefix.

The plasmid carrying *bla*_CMY-2_ (pCMY) assembled here was present alongside a *bla*_CTX-M-27_-carrying plasmid IncF (F2:A-:B-) in an E. coli ST10 isolate. This plasmid was of IncA/C2-ST3 type, 168 kbp in size, and carried resistance genes to aminoglycosides, sulfonamides, phenicols, and tetracyclines (Table 2; Fig. 6). The *bla*_CMY-2_ gene was flanked by IS*1380* transposase on one side and *blc*-*sugE* on the other, and this genetic environment is commonly associated with the presence and dissemination of *bla*_CMY-2_ genes universally. This plasmid (pCMY) had 92% coverage and 100% nucleotide identity with a *bla*_CMY-2_-carrying plasmid assembled recently from Salmonella enterica isolates from diseased pigs in the United States (accession number MK191845.1). Similarly, pCMY had 93% coverage and 99.97% nucleotide identity with respect to pUMNK88 (accession number HQ023862.1), which was among the first *bla*_CMY-2_-carrying plasmids to be isolated and assembled from E. coli isolates collected from diseased pigs in the United States in 2008. This plasmid was highly similar (97% coverage, 99% nucleotide identity) to another plasmid isolated from cow in the United States in 2002 (accession number FJ621588.1), suggesting that *bla*_CMY-2_-carrying IncA/C2 plasmids isolated from farm animals in the United States have remained relatively conserved over a long duration with the exception of a gain or loss of some AMR genes.

In addition to these ESBL-encoding plasmids, we also assembled a 59-kbp IncN plasmid carrying a *qnrB77* gene (Table 2; Fig. 6). This plasmid was present in a ST4981 isolate which also carried an ESBL-encoding gene (*bla*_CTX-M-15_) chromosomally. This plasmid also carried resistance genes to trimethoprim and aminoglycosides. The *qnrB77* gene was flanked by a complete and a truncated transposase of the IS*91* family of transposases (Table 2; Fig. 6).

Some of these plasmids (p1, p23, p33, and p65) also carried genes (*qacΔE*) that determine resistance to quaternary ammonium compounds. pCMY also carried the *sugE* gene that modulates resistance to quaternary ammonium compounds. Genes related to heavy metal resistance such as mercury (*merCDEPTR*), arsenic (*arsHB*), copper (*pcoES*), and tellurium (*terABCDWX*) resistance were also present on plasmids carrying *bla*_SHV-12_ and pCMY (*merCDEPTR* operon only). Plasmids carrying *bla*_CTX-M-55_ genes also carried tellurium resistance genes (*terABCDWX*). Additionally, all the plasmids assembled in this study carried mobility genes (*tra* set of genes) and genes that can aid in plasmid maintenance and stability. All of the IncFII and IncHI2 plasmids carried genes coding for at least one toxin-antitoxin system, e.g., the IncFII plasmids carried *pemI*-*pemK* genes, and all the IncHI2 and IncA/C2 plasmids carried *higA*-*higB* genes. Similarly, the *qnrB77-*carrying IncN plasmid also carried mobility genes (*tra*) and genes encoding proteins that aid in plasmid stability (*stbB*-*stbC* genes), antirestriction systems (*ardA*-*ardB* genes), and mutagenesis (*mucA*-*mucB* genes).

The comparison of these assembled plasmids with the PLSDB database resulted in the identification of several previously described plasmids with a high similarity (>80% coverage, and >98% nucleotide identity). To summarize, most of the plasmids carrying ESBL-encoding genes assembled in this study were similar to plasmids harbored on various *Enterobacteriaceae* and collected from various sources (animals, humans, and the environment) across different continents and shared the same molecular context around the genes of interest (*qnr* and *bla* genes) (Fig. 2 and 6). In contrast, we were not able to identify similar plasmids to the *bla*_CTX-M-15_-carrying IncFII (plasmid multilocus sequence type [pMLST], F48:A1:B49) and the *qnrB77*-carrying IncN plasmids found in this study.

We were also able to assemble 61 putative plasmids using these assembled plasmids as references (Table 3). Additionally, 1 *bla*_CTX-M-15_ was identified to be present on the chromosome of a ST4981 isolate. We also identified reference plasmids by conducting nucleotide BLAST searches in the GenBank database using contigs on which resistance genes were present. By mapping short reads onto these reference plasmids, we further identified that *bla*_CMY-2_ genes were present on a variety of IncI1-ST65, -12, -2, -20, -23, and -266 and IncI2 plasmids (Table 3). We also identified putative *bla*_CTX-M-27_-IncF- (F24:A-:B1), *bla*_CTX-M-55_-IncF- (F18:A-:B1), and *bla*_CTX-M-55_-IncI1-ST16 plasmids in our study (Table 3). Finally, we identified 6 *qnrB19*-ColE plasmids (sizes ranging between 2.8 and 3.2 kbp) and 2 *qnrS2*-IncQ2 plasmids (approximately 7.7 kbp in size) present as contigs in the draft E. coli isolates assembled (Table 3). Overall, by combining these different strategies, we were able to identify the AMR genes-plasmid/chromosome combinations for 85.1%, 91.7%, and 81.5% of the *bla*_CMY-2_, *bla*_CTX-M/SHV-12_, and PMQR genes, respectively.

**TABLE 3:** Characteristics of putative plasmids assembled in this study

Gene	Reference sequence GenBank accession no. (size [kbp])	pMLST	E. coli ST (no. of isolates)	Median coverage^*a*^ (range)	Median SNP difference (range)	Virulence gene(s)
*bla*_CMY-2_	MT816498.1 (168)	IncA/C2-ST3 (*n* = 53)	ST12 (20), ST100 (8), ST101 (6), ST10 (2), ST23 (2), ST410 (2), ST58 (2), ST744 (2), ST90 (2), ST127 (1), ST224 (1), ST6234 (1), ST69 (1), ST88 (1), ST93 (1), ST977 (1)	89.6 (72.3–97.2)	2 (0–18)	
	LC501512.1 (80)	IncI1-ST65 (*n* = 6)	ST10 (4), ST100 (1), ST23 (1)	95.5 (94.2–97.0)	10 (5–40)	*cia*
	MK191846.1 (99)	IncI1-ST12 (*n* = 5)	ST58 (2), ST75 (2), ST744 (1)	99.2 (95.5–99.9)	2 (0–157)	*cib*
	CP023356.1 (95)	IncI1-ST2 (*n* = 3)	ST101 (1), ST410 (1), ST58 (1)	98.8 (97.1–100)	3 (2–4)	*cia*
	CP027535.1 (101)	IncI1-ST20 (*n* = 1)	ST2025 (1)	92.9	5	*cib*
	CP009566.1 (95)	IncI1-ST23 (*n* = 1)	ST10 (1)	94.7	2	*cia*
	CP029976.1 (34)	IncI1-ST266 (*n* = 1)	ST766 (1)	99.9	14	
	CP043196.1 (65)	IncI2 (*n* = 3)	ST154 (1), ST23 (1), ST58 (1)	98.5 (97.9–98.6)	4 (4–10)	
	NA^*b*^	Unknown^*c*^ (*n* = 13)	ST10 (4), ST101 (2), ST12 (1), ST3057 (1), ST369 (1), ST410 (1), ST4373 (1), ST73 (1), ST90 (1)	NA	NA	NA
*bla*_CTX-M-14_	MT077889.1 (76)	IncF (F2:A-:B-) (*n* = 1)	ST10 (1)	91.7	0	*traT*
*bla*_CTX-M-15_	MT077880.1 (171)	IncF (F31:36:A4:B1) (*n* = 2)	ST617 (1), ST167 (1)	74.0–91.5	3–24	*traT*, *sitA*, *iucC*, *iutA*
	MT077882.1 (115)	IncF (F48:A1:B49) (*n* = 1)	ST744 (1)	96.9	0	*traT*
	NA	Unknown (*n* = 1)	ST410 (1)	NA	NA	NA
*bla*_CTX-M-27_	AP017621.1 (117)	IncF (F24:A-:B1) (*n* = 1)	ST1585 (1)	80.7	48	*hlyF*, *ompT*, *sitA*
*bla*_CTX-M-55_	MN158989.1 (128)	IncF (F18:A-:B1) (*n* = 3)	ST457 (2), ST744 (1)	90.1 (89.1–90.6)	5 (1–48)	*cma*, *cvaC*, *hlyF*, *iucC*, *iutA*, *ompT*, *sitA*, *traT*
	KX246268.1 (86)	Inc1-ST16 (*n* = 1)	ST101 (1)	98.9	0	
*bla*_SHV-12_	MT077886.1 (289)	IncHI2A-ST1 (*n* = 2)	ST10 (2)	95.1–95.2	8–9	*terC*
	NA	Unknown (*n* = 1)	ST641 (1)	NA	NA	NA
*qnrB19*	KY991369.1 (3)	ColE (*n* = 6)	ST10 (1), ST2161 (1), ST2496 (1), ST361 (1), ST5759 (1), ST93 (1)	95.8 (91.6–100)	0	
*qnrB2*	MT077886.1 (289)	IncHI2-ST1 (*n* = 2)	ST10 (2)	95.1–95.2	8–9	*terC*
	NA	Unknown (*n* = 2)	ST10 (1), ST540 (1)	NA	NA	NA
*qnrB77*	MT077883.1 (59)	IncN unknown (*n* = 2)	ST10 (1), ST100 (1)	90.7–91.9	2–4	
*qnrS1*	NA	Unknown (*n* = 2)	ST10 (1), ST641 (1)	NA	NA	NA
*qnrS2*	KT896500.1 (7.7)	IncQ2 (*n* = 2)	ST10 (1), ST101 (1)	99–100	0	
	NA	Unknown (*n* = 2)	ST10 (1), ST847 (1)	NA	NA	NA
*aac-(6′)-lb-cr*	MT077880.1 (170)	IncF (F31:36:A4:B1) (*n* = 2)	ST617 (1), ST167 (1)	74.0–91.5	3–24	*traT*, *sitA*, *iucC*, *iutA*
	NA	Unknown (*n* = 1)	ST410 (1)	NA	NA	NA

a Coverage was estimated using following formula: (size of reference − size of putative plasmid)/size of reference × 100.

b NA, not applicable.

c Unknown, plasmids/chromosomes carrying these genes were not identified for these isolates.

### Clonal and horizontal transmission of AMR.

The spread of ceftiofur and enrofloxacin resistance was bimodal. Both widespread dissemination of clones with specific E. coli*-*plasmid combinations as well as horizontal transmission of plasmids between genetically unrelated E. coli STs contributed to successful spread of AMR in the United States.

Fifty-four E. coli isolates belonging to 16 different STs harbored *bla*_CMY_-IncA/C3-ST3 plasmids (Table 3), which is an indicator of successful horizontal dissemination of these plasmids across multiple E. coli strains. Similarly, 6 E. coli-*bla*_CMY-2_-IncI1-ST65 isolates were of 3 different STs (ST10, ST23, and ST100) and 5 E. coli-*bla*_CMY-2_-IncI1-ST12 isolates were also of 3 different STs (ST58, ST75, and ST744) (Table 3). Similar to that for *bla*_CMY-2_ genes, *bla*_CTX-M_ genes were also present on a wide variety of plasmids and E. coli STs. For example, *bla*_CTX-M-55_ genes were present on 3 different plasmid types and present in 5 E. coli isolates of 4 different STs (ST165, ST457, ST744, and ST101) (Tables 3). *qnrB19* was the most prevalent PMQR gene and was located on ColE plasmids in 6 E. coli isolates, all of which were of different STs (Table 3). The details of the prevalence of *bla*_CTX-M_, *bla*_SHV-12_, and PMQR genes and their association with E. coli STs and plasmids are presented in Table 3.

There were also some clusters of genetically similar isolates having same ST-AMR genetic mechanism-plasmid type combinations, indicating clonal transmission of certain resistant bacterial clones throughout swine populations in the United States. Clusters of isolates with genetic distance between the next closest isolate being <100 single nucleotide polymorphisms (SNPs) and carrying genetic mechanisms of resistance relevant to this study are indicated in Fig. 1. The biggest examples of clonal transmission in our data set are ST100 E. coli isolates, 36 of which had chromosomal mutations in *gyrA83* and *parC80* and had a means median pairwise SNP distance (MPD) of 36 SNPs (range, 4 to 63 SNPs) (Fig. 1). Seventeen ST744 isolates had chromosomal mutations in *gyrA83*, *gyrA87*, *parC56*, and *parC80*, with an MPD of 115 SNPs (range, 10 to 203 SNPs) (Fig. 1). Twenty-two of the 23 ST12 and 6 of the 12 ST101 E. coli isolates carried *bla*_CMY-2_-IncA/C2-ST3 (MPD between these 22 ST12 isolates, 66 SNPs [range, 32 to 164 SNPs]; MPD between the 6 ST101 isolates, 70 SNPs [range, 31 to 87 SNPs]) (Fig. 1). It should be noted that some very close isolates harbored different AMR genes and plasmids. For example, in related ST10 isolates (MPD, 101 SNPs [range, 20 to 121 SNPs]), *bla*_CMY-2_ genes were present on IncA/C2-ST3, IncI1-ST23, and IncI1-ST65 plasmids (Fig. 1).

### Comparison with other isolates available on Enterobase.

**FIG 7:**
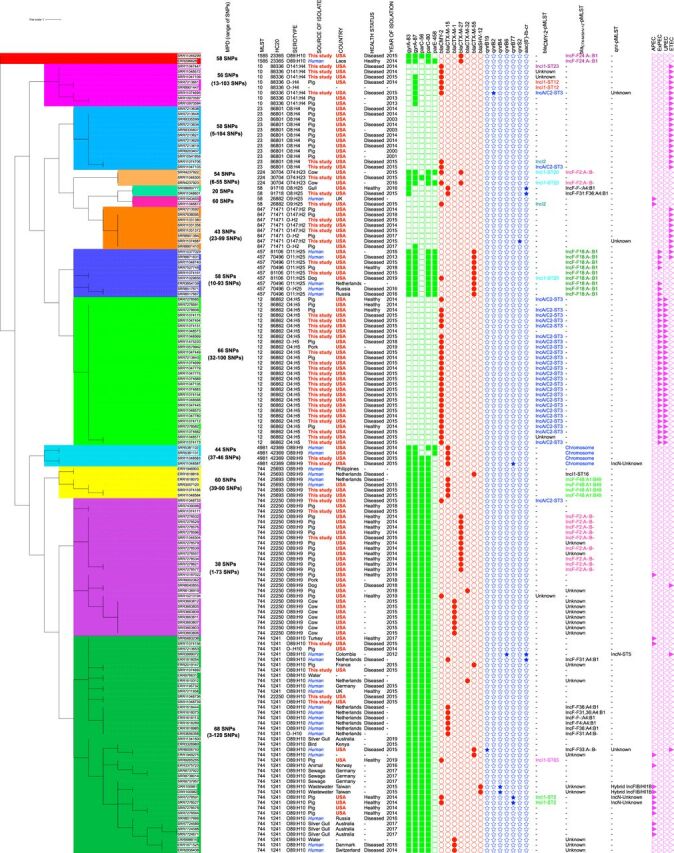
Maximum likelihood tree constructed using the core gene alignment of selected Escherichia coli isolates collected in this study and isolates available at Enterobase. E. coli isolates from Enterobase were selected by identifying those that were within 20 allelic differences (same HC20) of the isolates assembled in our study. This unrooted tree was created by mapping raw reads on E. coli K-12 substrain MG1655 (accession NZ_AJGD00000000.1), followed by extraction of SNPs and phylogenetic tree construction (GTR plus gamma substitution model, 1,000 bootstrap replicates) using RAxML version 8.0. Raw reads mapped onto 84.2% to 94.2% of the reference sequence E. coli K-12 substrain MG1655. Phylogenetic tree was constructed using 43,503 recombinant-free sites. Heat map shows presence of chromosomal mutations in quinolone resistance-determining regions (QRDRs) (green squares), plasmid-mediated quinolone resistance (PMQR) genes (blue stars), ESBL/pAmpC genes (red circles), and virulotypes (APEC, ExPEC, UPEC, and ETEC) (purple triangles). Median pairwise SNP distances (MPDs) were estimated using ST-specific references. Colored clusters represent groups of isolates with an SNP distance to the next closest isolate of less than 100. Unknown, plasmids/chromosomes carrying these genes were not identified for these isolates.

Some of the isolates of ST1581, -58, -457, -4981, and -744 in our study were found to be within 20 allelic differences of isolates collected from humans globally based on core genome multilocus sequence type (cgMLST) (Fig. 7). ST744 E. coli similar to those in this study were isolated from humans, pet animals, wild animals, farm animals, and environmental samples globally (Fig. 7). Specifically, these ST744 isolates were genetically similar (within 20 allelic differences) to those isolated from diseased and nondiseased humans in the Philippines, the Netherlands, the United States, Russia, Germany, the United Kingdom, Colombia, Denmark, and Switzerland. ST744 isolates from our study and the Enterobase collection consistently had mutations in quinolone resistance-determining regions (QRDRs) of *gyrA* and *parC* genes. Moreover, 2 ST744 isolates from our study were similar to isolates collected from diseased humans in the United States and the Netherlands (39 to 83 SNP differences) and also harbored *bla*_CTX-M-15_-carrying IncF (F48:A1:B49) plasmids, which have been described to be novel in this study (Fig. 7). Some of these ST744 isolates (including one from this study) were classified as avian pathogenic E. coli (APEC).

ST1581, ST457, and ST4981 isolates from our study also carried the same AMR genes, mutations, and plasmids as present in similar human isolates (Fig. 7). The ST457 isolate from this study was not classified into a virulotype, but other ST457 isolates in the Enterobase were classified as extraintestinal pathogenic E. coli (ExPEC). One of the APEC ST58 isolates was genetically similar to another isolate collected from a diseased human in the United Kingdom (SNP difference, 60).

Several other E. coli isolates were found to be genetically similar to those isolated from animals only. These consisted of ST10, ST23, ST224, ST847, and ST12. ST10, ST12, and ST847 were similar to those isolated from pork or healthy pigs in the United States, and ST10 and ST12 isolates from this study and the Enterobase collection had same genetic determinants of ceftiofur and enrofloxacin resistance. In this comparative analysis, 26 of 28 ST12 isolates were classified as ExPEC and uropathogenic E. coli (UPEC).

### Resistance determinants to other critical antimicrobials.

No carbapenem resistance genes were identified in our collection, but the *mcr-9* gene was present in 7 isolates belonging to 6 different STs. These isolates carried both the *mcr-9* gene and either a pAmpC, an ESBL, or a PMQR gene (Table 4). Descriptions of these isolates are presented briefly in Table 4. *mcr-9* was also present in two of the ESBL plasmids assembled in this study (Table 2).

**TABLE 4:** Characteristics of isolates carrying *mcr-9* genes

Isolate SRA accession no.	ST (serotype, phylotype)	Other AMR genes in the isolate [drug resistance]^*a*^	Virulotype	Plasmid replicons (pMLST results)
SRR11048580	540 (O9:H10, A)	*aac(6′)-IIc*, *aadA2b*, *aac(6′)-Ib3*, *aph(3″)-Ib*, *aph(6)-Id*, *aac(6′)Ib-cr* [AM]; *bla*_TEM-1b_ [PE]; *qnrB2* [FL]; *ere(A)*, *mdf(A)* [MA]; *sul1*, *sul2*, *sul3*, *dfrA12*, *dfrA19* [TS]; and *tet(A)*, *tet(M)* [TE]		IncHI2A (ST1), IncI1 (ST266), IncX1
SRR11045321	641 (O121:H10, B1)	*aac(6′)-Ib3*, *aac(6′)-IIc*, *aph(6′)-Id*, *aph(3″)-Ib*, *aadA2*, *aac(6′)-Ib-cr* [AM]; *bla*_TEM-1b_ [PE]; *qnrB2* [FL]; *ere(A)*, *mdf(A)* [MA]; *sul1*, *sul2*, *dfrA19* [TS]; *tet(D)*, *tet(B)* [TE]; *bla*_SHV-12_ [EC]	ETEC	IncFII (F13:F29:A-:B-), IncHI2A (ST1), p011
SRR11046298	1112 (O142:H27, A)	*aph(3″)-Ib*, *aph(6′)-Id*, *aph(3′)-Ia*, *aac(6′)-IIc*, *aadA2* [AM]; *bla*_TEM-1b_ [PE]; *qnrB2* [FL]; *ere(A)*, *mdf(A)* [MA]; *sul1*, *dfrA19* [TS]; *catA2* [PH]; *tet(B)*, *tet(D)* [TE]; and *bla*_SHV-12_ [EC]	ExPEC	IncFIB, IncFII (F2:A-:B25), IncHI2A (ST1)
SRR11047778	90 (O149:H19, C)	*aac(3)-VIa*, *aadA1*, *aadA2*, *aadA5*, *aph(3″)-Ib*, *aph(3′)-Ia*, *aph(6)-Id*, *armA* [AM]; *bla*_TEM-1b_ [PE]; *mph(E)*, *msr(E)*, *mdf(A)* [MA]; *sul2*, *dfrA1* [TS]; *floR* [PH]; *tet(A)*, *tet(B)* [TE]; and *bla*_CMY-2_ [EC]	APEC	Col156, IncA/C2, IncFIB, IncFIC, IncFII (F108:A-:B42), IncHI2A (ST unknown), IncI1 (ST12)
SRR11074163	101 (O82:H8, B1)	*aac(3)-VIa*, *aadA1*, *aph(3″)-Ib*, *aph(6)-Id* [AM]; *mdf(A)* [MA]; *sul1*, *sul2* [TS]; *floR* [PH]; *tet(A)* [TE]; and *bla*_CMY-2_, *bla*_CTX-M-55_ [EC]	APEC	IncFIB, IncFII (F24:A-:B1), IncHI2A (ST unknown), IncI1 (ST16), IncX1
SRR11074700	10 (O141:H4, A)	*aac(6′)-IIc*, *aadA2b*, *aph(3″)-Ib*, *aph(3′)-Ia*, *aph(6)-Id* [AM]; *bla*_TEM-1b_ [PE]; *qnrB2* [FL]; *ere(A)*, *mdf(A)* [MA]; *dfrA19*, *sul1*, *sul2* [TS]; *tet(B)*, *tet(D)* [TE]; and *bla*_CMY-2_, *bla*_SHV-12_ [EC]	ETEC, STEC	IncFIB, IncFII (F-:A-:B42), IncHI2A (ST1), IncI1 (ST65), IncI2, p011
SRR11074696	10 (O141:H4, A)	*aac(6′)-IIc*, *aadA2b*, *aph(3′)-Ib*, *aph(3′)-Ia*, *aph(6)-Id* [AM]; *bla*_TEM-1b_ [PE]; *qnrB2* [FL]; *ere(A)*, *mdf(A)* [MA]; *dfrA19*, *sul1*, *sul2* [TS]; *tet(B)*, *tet(D)* [TE]; and *bla*_CMY-2_, *bla*_SHV-12_ [EC]	ETEC, STEC	IncFIB, IncFII (F-:A-:B42), IncHI2A (ST1), IncI1 (ST65), IncI2, p011

a AM, aminoglycosides; PE, penicillins; FL, fluoroquinolones; MA, macrolides; TS, trimethoprim/sulfonamide; PH, phenicols; TE, tetracyclines; CO, colistin; EC, extended-spectrum cephalosporin.

## DISCUSSION

Whole-genome sequencing (WGS) of enrofloxacin- and ceftiofur-resistant E. coli revealed multiple determinants conferring resistance to these critical antimicrobials, which were present on a wide spectrum of STs recovered from the major swine-producing states in the United States. The use of both long- and short-read WGS technologies identified the genetic context of these resistance determinants for several isolates, suggesting determinants by which resistance may be spreading, such as plasmids carrying *bla*_CMY-2_, which previously established in *Salmonella* and E. coli populations circulating in food animals in the United States (14). We also assembled plasmids not previously described in isolates from swine or other food animals or retail meat in the United States.

Nearly 82% of the ceftiofur-resistant E. coli isolates carried a *bla*_CMY-2_ gene, which is consistent with findings in ceftiofur-resistant *Salmonella* isolates from diseased pigs collected during the same study period (26). However, 24 E. coli isolates in this study (including 2 isolates nonresistant to ceftiofur) carried *bla*_CTX-M_ or *bla*_SHV-12_ genes, indicating a much higher prevalence (18%) of *bla*_CTX-M_ in our isolates compared to that in ceftiofur-resistant *Salmonella* of swine origin (26). Still, our data suggest a more limited distribution of *bla*_CTX-M_ genes compared with reports in extended-spectrum cephalosporin-resistant E. coli isolates retrieved from swine in other upper-income countries in Europe and Asia such as Belgium (97.5%) and Hong Kong (87.5%) (32, 33). ESBL genes are responsible for extended-spectrum cephalosporin resistance globally in food animals (13). However, until the late 2000s, these genes were not found in food animal isolates collected in North America (34). In a study on E. coli isolates collected from diseased pigs at the University of Minnesota Veterinary Diagnostic Laboratory (UMN-VDL) in 2008, all ceftiofur-resistant isolates carried *bla*_CMY-2_ genes (35), whereas *bla*_CTX-M_-carrying E. coli in finishing pigs in the United States were first identified in 2011 (36). Since then, more recent studies have also reported the sporadic occurrence of *bla*_CTX-M_ genes in *Enterobacteriaceae* isolates of swine origin (including pork) in the United States (37, 38). Our study reinforces the results that ESBL genes might have been introduced in E. coli collected from pigs during the late 2000s and early 2010s (34).

Similar to that for ESBLs, the presence of PMQR genes [*qnr*, *aac(6′)-Ib-cr*] in food animal isolates in the United States had not been reported until recently (25, 26, 39, 40). There has also been an increase in PMQR genes in clinical *Salmonella* isolates from humans in the United States, and animal sources have been postulated to contribute to this surge (40). In this study, the presence of PMQR genes without additional QRDR mutations was sufficient to yield MIC values to the intermediate susceptibility levels (0.25 to 1 μg/ml) but not above (with the exception of 2 *qnrB19*-carrying isolates). This is consistent with previous reports suggesting PMQR genes such as *qnrB* and *qnrS* confer only lower level resistance to quinolones by inhibiting the binding of quinolones to DNA gyrase (41). However, these PMQR genes are known to supplement resistance caused by other determinants such as altered target enzymes (DNA gyrase), efflux pump activities, and deficiencies in outer membrane porin channels (42). The presence of PMQR genes in zoonotic bacteria and their clinical impact on both human and animal health should therefore be continuously monitored.

To the best of our knowledge, this is the first study to describe completely assembled plasmids carrying *bla*_CTX-M-14_, _-15_, _-27_, and _-55_, *bla*_SHV-12_, and *qnrB77* in E. coli isolates of swine origin in the United States. However, the close identities between some plasmids in this study and those already described in humans and animals globally indicate that the presence of ESBL genes in this isolate collection could be part of the pandemic expansion of ESBLs (13). *bla*_CTX-M-15_ and *bla*_CTX-M-14_ are considered the predominant ESBL genes in humans globally (13) and have also been identified in food animals, including pigs, worldwide (43–46). The plasmids carrying *bla*_CTX-M-15_ identified in our study were highly similar (98% coverage, >99% nucleotide identity) to other plasmids found in human E. coli isolates collected in the United States between 2009 and 2010 (47) (GenBank accession number CP009232), which were also described to have the same plasmid backbone as other ESBL gene-carrying plasmids reported worldwide (47). *bla*_CTX-M-14_-carrying plasmids identical to those found here were previously reported in human isolates in Hong Kong and characterized as an epidemic plasmid type (pHK01) (48) which has spread globally to other Asian (China, Vietnam, and South Korea) and European (Finland) countries (unpublished; GenBank accession numbers NC_013727.1, KU932024.1, KU987452.1, NC_013542.1, and NZ_CP018973.1). Families of insertion sequences (IS*26*, IS*Ecp9*, and IS*6*) that were part of the above-mentioned genetic contexts have also been demonstrated to be involved in transposing ESBL-encoding genes across plasmids and bacterial chromosomes (49).

IncA/C2 and IncI1 plasmids were the carriers of *bla*_CMY-2_ genes, which is consistent with previous studies conducted on Salmonella enterica and Escherichia coli from both farm animals and humans in the United States (20, 21, 26, 50, 51). The *bla*_CMY-2_-carrying IncI1-ST12, IncI1-ST65, and IncA/C2-ST3 plasmids assembled here were highly similar (>99% nucleotide identity) to those isolated from broiler E. coli in Japan (which, in turn, were highly similar to plasmids from European poultry) (52). This suggests that closely related bla_CMY-2_-carrying plasmids are disseminated globally in livestock.

In contrast to ESBL and pAmpC genes, *qnr* genes were also present on short (∼3 kbp) ColE and medium-sized (∼7 kbp) IncQ2 plasmids. ColE plasmids harboring *qnrB19* genes are among the most commonly isolated PMQR-plasmid combinations globally, and these have been isolated from bacteria of family *Enterobacteriaceae* from several animals, meat, and humans globally (25, 53–57). *qnrS2*-harboring IncQ2 plasmids have largely been found from aquatic sources such as a wastewater treatment plant in Israel (58) and rivers and aquaculture facilities in China (59), indicative of potential exchange of resistant bacteria between environmental sources and swine herds (21, 60).

It has been widely believed that the presence of plasmids in the absence of selective pressure imposes a metabolic fitness cost to the bacterial host (61). However, the fitness cost imposed due to plasmid carriage depends on the plasmid-bacterial host combination (62–64). There are several plasmid characteristics that facilitate plasmid stability in bacterial hosts: for example, IncF and IncI1-pMLST12 plasmids similar to those assembled here have a narrow host range and carry factors such as toxin-antitoxin systems which help maintain their stability in bacterial hosts in the absence of antimicrobial pressure (52, 65, 66). Similarly, IncHI2 plasmids similar to those assembled here carry genes which confer resistance to heavy metals, mutagenesis induction systems, etc., which can also contribute to their stability (67). IncA/C2, IncHI2, IncN, and IncQ2 plasmids have a broad host range and can survive in multiple bacterial species, including bacteria present in the environment, which can aid in their persistence and dissemination outside animal hosts (68, 69). In a study using *bla*_CMY-2_-carrying E. coli isolated from pigs in the United States, larger plasmids (IncI1 and IncA/C2 plasmids similar to the one assembled in this study) were shown to coexist without imposing metabolic costs to the bacterial host (62). Epidemic plasmids identical to those found in our study such as pHK01-like plasmids have been demonstrated to be conjugative *in vitro* (70). Hence, it can be postulated that these plasmids might aid in the successful establishment of ESBL and PMQR genes and persistence of AmpC beta-lactamases as mechanisms of cephalosporin and quinolone resistance in swine E. coli in the United States.

In addition to the above-mentioned properties, IncF plasmids also possessed virulence genes which can contribute to the fitness of bacterial clones inside mammalian hosts. For example, the *traT* gene related to serum resistance was found on every *bla*_CTX-M_-harboring IncF plasmid, except for IncF (F24:A-:B1), and this gene has been consistently associated with urinary tract infections and sepsis in humans (71–74). Similarly, *hlyF* (hemolysin), *iutA* (iron uptake), and *ompT* (outer membrane protease) genes present on some of the *bla*_CTX-M_-IncF plasmids are very commonly found in avian pathogenic E. coli (75). *iucC* (aerobactin synthesis), *iutA*, and *ompT* genes also play key roles in the pathogenesis of severe extraintestinal infections in humans (76–78). The presence of virulence factors and antimicrobial resistance genes on epidemic IncF plasmids have previously contributed to global domination of E. coli ST131 clones (30) and can potentially aid in the successful dissemination of emerging E. coli lineages identified in this study.

The main enrofloxacin-resistant swine-specific ST identified in this study was ST100, which is associated with porcine enterotoxigenic infections and has spread clonally throughout the U.S. swine population (79). Enrofloxacin was approved to treat swine enteric infections in the United States in 2012 (2), and the association of enterotoxigenic ST100 E. coli with enrofloxacin resistance might be of concern for swine health because of the potential decrease in clinical efficacy of enrofloxacin in treating scours due to bacterial resistance.

Many of the isolates in this study can be considered to be “high-risk” clones. These clones are characterized by global dissemination, ease of transmission from host to host, disease-causing abilities, and acquisition of genetic characteristics that provide a competitive advantage over other bacterial clones, such as virulence factors, epidemic plasmids, and antimicrobial resistance genes (30). One outstanding example of a high-risk clone in our database is the fluoroquinolone-resistant ST744 isolates which were also closely related to isolates collected globally from multiple host species and the environment as well as from diseased humans. ST744 isolates belonged to phylogroup A and E. coli in this phylogroup are not as virulent as those belonging to phylogroups B2 and D2 (80). Nonetheless, ST744 has been sporadically associated as a disease-causing agent carrying resistance to critical antimicrobials such as colistin and carbapenem from human patients worldwide (81–83). The global distribution of fluoroquinolone-resistant ST744 clones should be worrisome, as fluoroquinolones are critical antimicrobials for treating systemic infections in humans (84). Fluoroquinolone-resistant ST410 was also present in our data set. Recently, Roer et al. described the emergence of ST410 (phylogroup A) high-risk clones globally, indicating that the clones of lowly virulent E. coli are also capable of widespread dissemination and carriage of antimicrobial resistance genes (85). More experiments and clinical studies are needed to determine the true pathogenicity and fitness of ST410 and ST744 high-risk clones.

ESBLs have been associated with pandemic ST131 E. coli in humans (86). However, in this study, only one ST131 isolate was identified, and it was susceptible to both antimicrobial classes under study but was classified as ExPEC and APEC. Manges et al. (87) recently published a review of the most prevalent global ExPEC lineages, and 12 of the top 20 ExPEC E. coli STs listed in this review were present in the U.S. swine E. coli isolates (ST131, ST69, ST10, ST73, ST410, ST12, ST127, ST167, ST58, ST88, ST617, and ST23) (87). In a study conducted on clinical E. coli isolates from a north California community, 47% of ExPEC strains consisted of ST127, -73, -69, -10, -12, and -88, which were all present in our study (88). Isolates of several STs in this study carried *bla*_CTX-M_-epidemic plasmids similar to IncF (F31:F36:A4:B1) and IncF (F2:A-:B-), had mutations in QRDRs conferring fluoroquinolone resistance, and were classified into several virulotypes of public health concern (Shiga toxin-producing E. coli [STEC], ExPEC, APEC, and UPEC), presenting further evidence of the presence of potentially high-risk zoonotic clones in the swine population in the United States.

The colistin resistance gene (*mcr-9*) was recently described for the first time in an S. enterica serovar Typhimurium isolate collected from a human patient in Washington state and was able to confer colistin resistance to E. coli isolates cloned with this gene (89). Tyson et al. further evaluated the presence of this gene in *Salmonella* and E. coli collected from animal meat in the United States and found that this gene was present on large IncHI2 plasmids similar to those found here or integrated into bacterial chromosomes (90). Tyson et al. also found that *mcr-9-*carrying bacterial isolates were all susceptible to colistin (90); hence, the clinical relevance of this gene on human health is still debatable. Regardless of clinical impact, colistin has never been used in swine in the United States; therefore, the presence of the *mcr-9* gene in swine could be an indicator of the complex transmission dynamics of resistant determinants across different ecosystems and/or a coselection of resistant determinants due to the use of other unrelated antimicrobials.

Several considerations must be accounted for when interpreting these results. An association between antimicrobial use and presence of these resistance genes cannot be established due to the lack of information on the use of antimicrobials. Also, the public health implications of our findings could be limited by the removal of diseased pigs, such as the ones from which these resistant and potentially zoonotic STs were retrieved, from the food chain.

### Conclusions.

We have identified and characterized a wide range of genetic determinants of resistance to some critically important antimicrobial classes in swine clinical E. coli isolates, some of which had never been described in isolates of animal origin in the United States. We also highlighted the presence of high-risk clones and epidemic plasmids in swine E. coli with a potential to negatively impact human and animal health.

## MATERIALS AND METHODS

### Description of isolates.

A total of 211 E. coli isolates recovered from diseased pigs at the University of Minnesota Veterinary Diagnostic Laboratory (UMN-VDL) between 2014 and 2015 were included in this study. E. coli isolates available at the UMN-VDL infectious agent repository were classified as ceftiofur non-wild type (MICs ≥ 2 μg/ml) and enrofloxacin non-wild type (MIC ≥ 0.25 μg/ml) (91). These MIC values were routinely estimated during the processing of diagnostic submissions and were based on the results of broth microdilution tests performed using Clinical and Laboratory Standards Institute guidelines (92). For ease of interpretation, “non-wild-type” and “wild-type” isolates are referred to as “resistant” and “susceptible,” respectively. All the ceftiofur- and/or enrofloxacin-resistant isolates available at the UMN-VDL infectious disease repository collected between 2014 and 2015 were selected for this study, and a random selection of susceptible isolates from the same period was used for comparative purposes. Of these 211 isolates, 110 were enrofloxacin resistant and 106 were ceftiofur resistant, with 41 isolates being resistant to both ceftiofur and enrofloxacin. Forty-six isolates susceptible to both antimicrobials were added to assess the presence of resistance genes and chromosomal mutations in susceptible isolates. Only one isolate per farm was selected in order to avoid duplicity of potentially identical clones circulating in the same farm.

### Short-read sequencing and *in silico* typing of E. coli isolates.

Isolates were first subjected to short-read sequencing using Illumina HiSeq 2500 (2 × 125 bp). The mean phred scores of raw reads was greater than 30 for all isolates. Raw reads were then trimmed using Trimmomatic version 0.39 (settings: sliding window mode; number of bases to average across, 4; average quality required, 20) (93). The raw reads were uploaded to and assembled using the QAssembly version 3.61 pipeline provided by the Enterobase webserver (94). This pipeline assembles draft genomes using Spades version 3.9.0 (95). The assemblies were further polished using BWA version 0.7.12 (96) to align reads back onto the assemblies, and these assemblies were polished using consensus bases or indels using bcftools version 1.2 (97). The assemblies were then passed for downstream analyses only if they meet the following criteria: number of bases, 3.7 to 6.4 Mbp; *N*_50_, >20 kb; number of contigs, <800; proportion of N’s, <3%; species assignment using Kraken, >70% contigs. The assembly statistics are as follows: average coverage, 107.3× (range, 52 to 405×); average *N*_50_, 135,520.4 bp (range, 40,154 to 340,030 bp); average size, 5.26 Mbp (range, 4.62 to 5.94 Mbp); and average number of contigs (>200 bp size), 209.4 (range, 71 to 392).

Draft genomes, assembly statistics, serotyping, phylotyping, and cgMLST results for these E. coli isolates were downloaded from the Enterobase webserver. Draft genomes were uploaded to the Center for Genomic Epidemiology (CGE) webserver to identify multilocus sequence type (MLST version 2.0.4) (98), acquired resistance genes and chromosomal mutations in quinolone resistance-determining regions (QRDRs) of *gyrA*, *gyrB*, *parC*, and *parE* genes (ResFinder version 3.2) (99), plasmid multilocus sequence type (pMLST version 0.1.0) (100), plasmid replicon type (Plasmid Finder version 2.0.1) (100), and virulence factors (virulence finder version 2.0) (101). A minimum nucleotide identity of 90% and minimum length of 60% compared to reference sequences were used as thresholds for classifying virulence and resistance genes. Draft genomes were annotated using PROKKA (version 1.13) (102).

Isolates were classified into virulotypes based on the virulence genes present as follows: (a) enterotoxigenic E. coli (ETEC) if any of the heat stable toxin genes (*sta*, *stb*, or *astA*) or heat-labile toxin gene (*ltcA*) were present, (b) Shiga toxin-producing E. coli if Shiga toxin genes (*stx*) were present, (c) extraintestinal pathogenic E. coli (ExPEC) if two or more of the genes *papA-papC*, *sfa-focDE*, *afa/draBC*, *kpsMII*, or *iutA* were present (103), (d) avian pathogenic E. coli (APEC) if all of the following genes, *iutA*, *hlyF*, *iss*, *iroN*, and *ompT*, were present (75), and (e) uropathogenic E. coli if three or more of the genes *chuA*, *fyuA*, *vat*, and *yfcV* were present (104).

### Phylogenetic analysis.

For the phylogenetic analysis, raw reads were first mapped to a reference genome (E. coli strain K-12 substrain MG1655; accession NZ_AJGD00000000.1), and full gene alignments were assembled using snippy (default values: minimum mapping quality, 60; minimum coverage, 10; version 4.4.5) (105). ClonalframeML (default values, version 1.12) was used to detect recombinant regions from these full gene alignments (106), which were subsequently masked by using maskrc-svg (version 0.5) (107). Single nucleotide polymorphisms (SNPs) were extracted from these recombination-masked alignments using snp-sites (version 2.5.1) (108), and pairwise SNP distances were estimated using snp-dists (version 0.7.0) (108). Maximum likelihood trees were then built using a general time-reversible (GTR) with gamma substitution model through RAxML (version 8.0) (109). Support for nodes on trees was assessed using 1,000 bootstrap replicates, and the phylogenetic tree was made and genomic features were annotated using iTOL (version 4.0) (110). Based on this analysis, we identified STs with SNP distances of less than 100 between at least 2 isolates and ran an ST-specific phylogenetic analysis for these STs using the following reference sequences: ST10 (NZ_AJGD00000000.1), ST12 (CP010151.1), ST23 (CP007491.1), ST58 (CP043744.1), ST88 (CP031546.1), ST90 (CP020520.1), ST100 (CP002729.1), ST101 (CP024821.1), ST224 (CP035339.1), ST410 (CP031231.1), ST457 (CP024826.1), ST641 (CP046000.1), ST744 (GCF_001682305), ST847 (CP010344.1), and ST4981 (CP017980.1). For each ST-specific analysis, the chromosomal sequence with maximum genome coverage available at the RefSeq database was selected as a reference. The steps for SNP estimation and measurement of SNP distances were repeated for each ST-specific analysis as mentioned above for E. coli strain K-12 substrain MG1655 as the reference. In the text and figures, median pairwise SNP distances (MPDs) and the ranges of SNPs mentioned are based on these ST-specific analyses.

We downloaded raw reads of all isolates available at Enterobase which belonged to the same cgMLST clusters (defined by a maximum difference of 20 alleles [“HC20”]) as the ceftiofur- or enrofloxacin-resistant isolates in our study. Phylogenetic analyses using E. coli K-12 substrain MG1655 and ST-specific references and typing (virulence genes, AMR genes, phylotyping, pMLST, plasmid analysis, and serotyping) were repeated using the same steps as mentioned above for comparing our isolates with similar isolates (same HC20) available at Enterobase.

Detailed results of serotyping, phylotyping, MLST, cgMLST, virulence factors, antimicrobial resistance genes, plasmid replicon typing, and associated metadata are available in Tables S1 and S2 in the supplemental material.

10.1128/mSphere.00990-20.2 TABLE S2 Detailed virulence, antimicrobial resistance, and typing metadata of isolates available on Enterobase that were within 20 allele differences (as per cgMLST schema provided at Enterobase) of isolates in this study. Download Table S2, XLSX file, 0.1 MB. Copyright © 2020 Hayer et al. 2020 Hayer et al. This content is distributed under the terms of the Creative Commons Attribution 4.0 International license.

### Assembly of plasmids using long-and short-read sequencing.

Additionally, long-read sequencing was performed on a subset of isolates carrying *bla*_SHV-12_, *bla*_CTX-M_, *bla*_CMY-2_, and *qnrB77* genes in the analysis described above using Pacific Biosciences (PacBio) RSII technology (SMRT Cell 1M v3). This subset was selected on the basis of presence of ESBL genes to represent all genomic contexts around these genes (available from contigs assembled on short reads). Long reads were first corrected for errors using LoRDEC (version 0.9) (111). Unicycler (version 0.4.7) (112) was used to obtain *de novo* hybrid assemblies of these isolates using both long and short reads, and assemblies were visualized using Bandage (version 0.8.1) (113). Complete plasmid genomes (here referred to as “assembled plasmids”) were uploaded to the ISsaga webserver (114) for identification of insertion sequences and to the CGE webserver to perform analyses as mentioned above. The assembled plasmids were also analyzed against a blast database of reference plasmids available at the PLSDB webserver (115) to identify closely related plasmids also carrying antimicrobial resistance genes of interest (ESBL and PMQR). Plasmid sequences with a query coverage of >80% and nucleotide identity >90% were downloaded, and the top five closely related plasmids and genomes to each of the ones found here were visually compared using BRIG (version 0.95) (116).

### Assembly of putative plasmids.

“Putative plasmids” were also assembled using these assembled plasmids. This was performed by mapping short reads of isolates carrying the same ESBL, PMQR, or pAmpC gene as the assembled plasmids carrying the corresponding genes. The mapping was conducted using snippy (version 4.4.5) with the values mentioned above. Pileups were generated after mapping short reads using SAMtools (version 1.10) with mapping quality capped at 60 (97). Pileups were then converted to fasta format using Galaxy tools 1.0.2 (117).

In the cases where raw reads did not sufficiently map to the assembled plasmids (query coverage of putative plasmid <70% compared to assembled plasmids), we identified the closest “reference plasmids” by doing a BLASTN search of the contigs carrying AMR genes on the NCBI server and assembled putative plasmids by mapping short reads onto these reference plasmids. Contigs from our study had >90% query coverage compared to these reference plasmids, with a nucleotide identity of >99%. We also confirmed that plasmids in our study were indeed similar to these reference plasmids by comparing the pMLST results for these reference plasmid sequences, putative plasmids assembled, draft E. coli genomes, and the contigs carrying these AMR gene in draft genomes. Analysis of pMLSTs for reference plasmids, putative plasmids, and draft E. coli sequences provided complete information on the sequence type of the plasmids. The pMLST results for just the contigs carrying AMR genes provided only partial matches, with the exception of short plasmids. These assembled and putative plasmids were then annotated, and genomic features such as virulence factors and insertion sequences were identified as mentioned above. Putative plasmids were also assembled for the isolates downloaded from Enterobase using same methods as described above.

### Data availability.

Short reads generated during this project have been submitted at NCBI GenBank under BioProject accessions PRJNA605257, PRJNA605064, and PRJNA604903. Complete plasmid sequences have been submitted at GenBank under accession numbers MT077880, MT077881, MT077882, MT077883, MT077884, MT077885, MT077886, MT077887, MT077888, MT077889, and MT816498.
